# Primary pulmonary meningioma: a case report

**DOI:** 10.1093/jscr/rjae406

**Published:** 2024-06-04

**Authors:** Manjinder Kaur Pannu, Jonas Peter Ehrsam, Othmar Markus Schöb, Ilhan Inci

**Affiliations:** Department of Basic and Clinical Sciences, University of Nicosia Medical School, Makedonitissas Avenue, CY-2417, Nicosia, Cyprus; Klinik Hirslanden Zürich, Thoracic Surgery Clinic, 8032, Zürich, Switzerland; Klinik Hirslanden Zürich, Thoracic Surgery Clinic, 8032, Zürich, Switzerland; Department of Basic and Clinical Sciences, University of Nicosia Medical School, Makedonitissas Avenue, CY-2417, Nicosia, Cyprus; Klinik Hirslanden Zürich, Thoracic Surgery Clinic, 8032, Zürich, Switzerland

**Keywords:** thoracic surgery, pulmonary meningioma, oncological resection

## Abstract

An asymptomatic 68-year-old woman, with a history of breast cancer 19 years ago, was unexpectedly found to have primary pulmonary meningioma during medical evaluation. This discovery is exceedingly rare, with only about 70 cases reported worldwide. Following uncomplicated surgical removal of the mass, the patient was discharged in good health on the third day after the procedure. Notably, initial analysis of a frozen tissue sample indicated hamartoma, but subsequent immune-histochemical pathological examination confirmed the presence of meningioma. Given the uncommon nature of this tumor, it is essential to report such cases to raise awareness about pulmonary meningioma as a potential cause of solitary lung nodules. This awareness can help prevent unnecessary chemotherapy or surgical interventions.

## Introduction

Meningioma, the most common primary central nervous system (CNS) tumor, characteristically arises from the meninges of the brain and spinal cord and accounts for ~37.6% of all cases [1, 2]. Characteristically, it arises from the meningeal layers of the brain or the spinal cord [3, 4]. However, primary pulmonary meningioma (PPM) is incredibly rare, with ~70 cases being reported worldwide [5].

Herein, we report such a case of PPH.

## Case report

**Figure 1:**
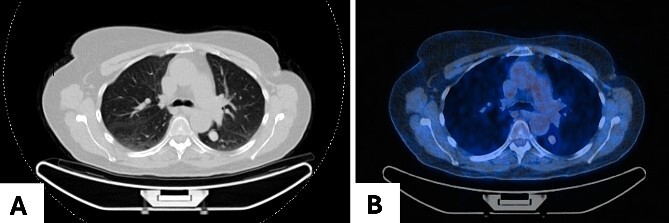
Pulmonary meningioma in segment VI of the left lower lobe in axial view in (A) CT scan showing a well-circumscribed/bordered homogenous, solid, noncalcified lesion; (B) PET-CT scan showing minimal metabolic activity.

A 68-year-old woman was referred to our clinic for surgical evaluation after abnormal findings on a computed tomography (CT) scan (Fig. 1A). She had a history of breast cancer for which she underwent surgical resection and local lymphadenectomy in 2005. Despite being asymptomatic, and nearly two decades later in 2024, her CT scan revealed a well-defined 1.5-cm lesion in segment VI of the left lower lobe. A subsequent PET-CT scan (Fig. 1B) showed minimal metabolic activity in this area, and no further abnormalities. Initially, the lesion was suspected to be a hamartoma, with lung carcinoma considered as a potential alternative diagnosis.

During the interdisciplinary meeting, various options were discussed, including direct diagnostics, therapeutic resection, or simply monitoring the dynamics of the lesion through radiological means. Among these options, the patient decided to undergo surgical resection to obtain clarity. The diagnostics operation was scheduled as a robot-assisted thoracoscopy for March 2024.

During surgery, the lesion was neither direct nor indirectly visible; therefore, the decision for a direct oncological resection of the segment VI along with systematic lymphadenectomy was performed. The entire surgical procedure lasted 53 minutes, with minimal intraoperative blood loss totaling only 30 mL. The recovery after surgery proceeded without any issues, and the patient was released from the hospital on the third day postoperation, without encountering any complications and in a generally favorable state of health.

**Figure 2:**
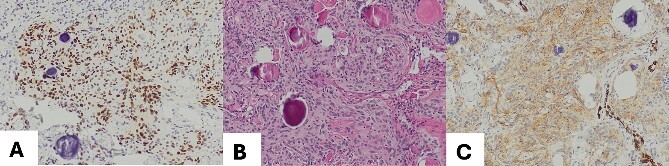
(A) Meningothelial cells with some psammoma corpuscles; magnification 100×; (B) Image EMA: immunohistochemical positivity for EMA, and magnification 100×; (C) progesterone image: immunohistochemical positivity for progesterone; magnification 100×.

The excised tumor measured 1.5 cm in its largest dimension. Final pathology confirmed negative margins. Notably, while the initial intraoperative diagnosis based on frozen section analysis indicated a hamartoma, the subsequent comprehensive pathological evaluation revealed the presence of a transitional type of pulmonary meningioma with evidence of numerous psammoma corpuscles on microscopic examination. Further analysis showed that the tumor tested positive for epithelial membrane antigen (EMA) and progesterone (PR), with a low proliferation rate (<5%), confirming the diagnosis of a pulmonary meningioma (Fig. 2). There were no signs of abnormal cell features or increased cell division rate, which would indicate an atypical or malignant meningioma.

## Discussion and conclusions

PPM is an exceedingly rare finding, characterized by the presence of meningioma in the lungs without any concurrent meningioma in the CNS. Globally, only about 70 cases have been reported over the past four decades [6]. Most PPMs are benign, typically ranging from 0.4 to 6.5 cm in diameter, although a handful of malignant cases have been documented [6], with sizes ranging from 1.5 to 15 cm [7].

As in our reported case, PPMs often manifest asymptomatically, typically appearing as incidental solitary lesions on imaging studies [7, 8]. However, some cases may present as ground-glass density nodules [7] or multiple solid nodules, with or without nonspecific symptoms [9].

Accurate diagnosis is crucial due to reported high rates of misdiagnosis, which can lead to unnecessary extensive pulmonary resections and chemotherapy [8].

Since pulmonary metastases from benign meningiomas are exceedingly rare but have been documented [10], confirming a diagnosis of PPM involves ruling out cerebral meningiomas along with obtaining pathological confirmation. In our patient, primary cerebral meningioma was excluded using postoperative brain MRI. Immuno-histological staining is often utilized to verify diagnosis and distinguish PPMs from other lung tumors histologically. Characteristic markers include positivity for somatostatin receptor 2 (SSTR2A), EMA, and PR [11], as our case which was positive for both, EMA and PR. Additional negativity for signal transducer and activator of transcription (STAT6) and SOX may be seen supportively [11]. Benign PPMs display histology and immunophenotypes consistent with their primary intracranial counterpart [12]. However, there are no histological features specific to malignant PPMs that would facilitate differentiation between the two tumor types. Therefore, it is advisable to rely on the diagnosis and grading of CNS meningiomas in cases of malignant PPM [4].

The exact cause of PPM remains unclear, but it is proposed to originate from the proliferation of ectopic embryonic nests of arachnoid cells, minute pulmonary meningothelial-like nodules [13, 14], or pluripotent subpleural mesenchyme [15].

Surgical resection is the primary treatment for PPM, with wedge [7] or segmental resection being common approaches. Intraoperative pathological examination is essential for determining the required scope of surgery. The prognosis for PPM is generally excellent, with cases of no recurrence reported even after 20 years following resection with tumor-free margins [12, 15].

## Data Availability

The datasets used are available from the corresponding author on reasonable request.
